# Identification and Analysis of Candidate Genes Associated with Yield Structure Traits and Maize Yield Using Next-Generation Sequencing Technology

**DOI:** 10.3390/genes15010056

**Published:** 2023-12-29

**Authors:** Bartosz Nowak, Agnieszka Tomkowiak, Aleksandra Sobiech, Jan Bocianowski, Przemysław Łukasz Kowalczewski, Julia Spychała, Tomasz Jamruszka

**Affiliations:** 1 Smolice Plant Breeding Ltd., IHAR Group, Smolice 146, 63-740 Kobylin, Poland; nowak@hrsmolice.pl; 2 Department of Genetics and Plant Breeding, Poznań University of Life Sciences, Dojazd 11, 60-632 Poznań, Poland; aleksandra.sobiech@up.poznan.pl (A.S.); julia.spychala@up.poznan.pl (J.S.); tomasz.jamruszka@up.poznan.pl (T.J.); 3 Department of Mathematical and Statistical Methods, Poznań University of Life Sciences, Wojska Polskiego 28, 60-637 Poznań, Poland; jan.bocianowski@up.poznan.pl; 4 Department of Food Technology of Plant Origin, Poznań University of Life Sciences, Wojska Polskiego 31, 60-624 Poznań, Poland; przemyslaw.kowalczewski@up.poznan.pl

**Keywords:** maize, next-generation sequencing (NGS), association mapping, yield structure traits, yield, SNP markers, SilicoDArT markers

## Abstract

The main challenge of agriculture in the 21st century is the continuous increase in food production. In addition to ensuring food security, the goal of modern agriculture is the continued development and production of plant-derived biomaterials. Conventional plant breeding methods do not allow breeders to achieve satisfactory results in obtaining new varieties in a short time. Currently, advanced molecular biology tools play a significant role worldwide, markedly contributing to biological progress. The aim of this study was to identify new markers linked to candidate genes determining grain yield. Next-generation sequencing, gene association, and physical mapping were used to identify markers. An additional goal was to also optimize diagnostic procedures to identify molecular markers on reference materials. As a result of the conducted research, 19 SNP markers significantly associated with yield structure traits in maize were identified. Five of these markers (28629, 28625, 28640, 28649, and 29294) are located within genes that can be considered candidate genes associated with yield traits. For two markers (28639 and 29294), different amplification products were obtained on the electrophorograms. For marker 28629, a specific product of 189 bp was observed for genotypes 1, 4, and 10. For marker 29294, a specific product of 189 bp was observed for genotypes 1 and 10. Both markers can be used for the preliminary selection of well-yielding genotypes.

## 1. Introduction

Biological progress in modern plant breeding is defined as the development of new genotypes with traits relevant to agricultural practice [1,2]. These traits are associated with plant productivity and health, the suitability of produced materials for processing, as well as meeting the expectations of consumers (food) and non-consumable material users (e.g., cellulose-based resources). Maize breeding is aimed at developing high-yielding hybrid varieties [3,4,5]. This progress increasingly relies on the application of genomics and genetic engineering advancements [6].

Maize breeding worldwide is based on a wide range of research techniques in molecular genetics, primarily in two areas. The first is making selection decisions based on DNA nucleotide sequence analysis, and the second is expanding genetic variability in breeding populations through genetic modifications, primarily by developing plant organisms with foreign species genes [7,8]. This has not only created attractive prospects for achieving biological progress but also opened new possibilities for the utilization of not only maize but also other crops [5]. 

The introduction of molecular analysis for genetic markers has enabled the development of selection methodologies based on genetic markers—known as marker-assisted selection (MAS). The scope of applying this methodology is clearly dependent on the progress of knowledge about the genome of a given species [9,10]. The breakthrough in genomics was the completion of the first stage of sequencing the human genome and the announcement in February 2001 of two publications, published in “*Nature*” and “*Science*”, describing its organization [11,12]. This achievement has opened up a wide range of possibilities for characterizing the genomic sequences of crop plants [13,14,15,16]. The identification of genome sequences has revealed the presence of a vast number of differences in the studied genomes, with the majority being single-nucleotide polymorphisms (SNPs). They have been used to create a tool for a global genome analysis—the so-called SNP microarray—enabling the simultaneous determination of genotypes at thousands of loci [17]. SNP microarrays have become a key tool in the search for unknown polymorphisms (mutations) responsible for the occurrence of genetic diseases (e.g., monogenic) or predisposition to their development (e.g., complex diseases), as well as the phenotypic variability of production traits. The procedure based on SNP microarrays is commonly referred to as a GWAS (genome-wide association study) and is widely used primarily in human genomics and increasingly in animal and plant genomics, including maize [18].

To identify trait markers, it is necessary to have a large number of markers that densely and uniformly cover the genome. The density of this coverage depends on the linkage disequilibrium, which is species- and trait-dependent. As a result, such studies require markers obtained by next-generation sequencing methods such as GBS [17] or DArTseq and relatively large computational power. Two approaches are distinguished in association mapping: candidate gene association and genome-wide association studies (GWASs). Conducting a GWAS involves searching for trait–marker associations in the entire genome, assuming that there are markers showing linkage disequilibrium within the gene regulating the expression of a given trait [19]. Initially, association mapping performed in maize [20] did not consider population structure. This false-positive association was filtered out by the study of Pritchard, who included population structure in his maize study [21].

With the advancement of efficient marker methods and the availability of statistical software (Genstat 23), the number of analyzed species has increased, and DNA markers identified by this method are currently used in breeding practice [22,23,24]. Association mapping has proven useful for identifying the markers of traits whose quantitative loci explain a significant portion of trait variation [25]. However, this method has limited application for complex traits with weak effects of individual loci [26]. 

For several years, maize breeding worldwide has been supported by useful molecular markers. Many authors have stated in their publications that marker-assisted breeding accelerates yield growth not only in the USA but also in other countries, offering enormous potential to enhance maize productivity and germplasm value [27,28].

Therefore, the aim of the present study was to identify new markers linked to candidate genes determining grain yield using next-generation sequencing, gene association, and physical mapping, as well as to optimize the diagnostic procedures for the identification of 19 selected molecular markers.

## 2. Materials and Methods

### 2.1. Plant Material

The plant material included 64 inbred lines, 122 F_1_ hybrids, and 20 reference genotypes of maize (both high-yielding and low-yielding). The plant material was derived from Hodowla Roślin Smolice Sp. z o.o. Grupa IHAR (51°42′12″ N 17°10′10″ E) and Małopolskia Hodowla Roślin Sp. z o.o. (50°58′17″ N 16°55′50″ E). Part of the analyzed lines were flint grain lines of three different origins: F_2_ (a group related to the F_2_ line, bred at INRA in France from the Lacaune population), EP1 (a group related to the EP1 line, bred in Spain from the population derived from the Pyrenees), and German Flint. The second part of the plant material was dent-type kernels derived from various groups of origin from the United States: Iowa Stiff Stalk Synthetic (BSSS), Iowa Dent (ID), and Lancaster.

### 2.2. Methods

#### 2.2.1. Open Field Experiment

This study was based on a total of 188 maize inbred and hybrid lines (G01.01, G01.02, G01.03, G01.04, G01.05, G01.06, G01.07, G01.08, G01.09, G01.10, G01.11, G01.12, G01.13, G01.14, G01.15, G01.16, G01.17, G01.18, G01.19, G01.20, G01.21, G02.01, G02.02, G02.03, G02.04, G02.05, G02.06, G02.07, G02.08, G02.09, G02.10, G02.11, G02.12, G02.13, G02.14, G02.15, G02.16, G02.17, G02.18, G02.19, G02.20, G02.21, G03.01, G03.02, G03.03, G03.04, G03.05, G03.06, G03.07, G03.08, G03.09, G03.10, G03.11, G03.12, G03.13, G03.14, G03.15, G03.16, G03.17, G03.18, G03.19, G03.20, G03.21, G04.01, G04.02, G04.03, G04.04, G04.05, G04.06, G04.07, G04.08, G04.09, G04.10, G04.11, G04.12, G04.13, G04.14, G04.15, G04.16, G04.17, G04.18, G04.19, G04.20, G04.21, G05.01, G05.02, G05.03, G05.04, G05.05, G05.06, G05.07, G05.08, G05.09, G05.10, G05.11, G05.12, G05.13, G05.14, G05.15, G05.16, G05.17, G05.18, G05.19, G05.20, G05.21, G06.01, G06.02, G06.03, G06.04, G06.05, G06.06, G06.07, G06.08, G06.09, G06.10, G06.11, G06.12, G06.13, G06.14, G06.15, G06.16, G06.17, K037, K038, K039, K040, K041, K042, K043, K044, K045, K046, K047, K048, K049, K050, K051, K052, K053, K054, K055, K056, K057, K058, K059, K060, K061, K062, K063, K064, K065, K066, K067, K068, K069, K070, K071, K072, K073, K074, K075, K076, K077, K078, K079, K080, K081, K082, K083, K084, K085, K086, K087, K088, K089, K090, K091, K092, K093, K094, K095, K096, K097, K098, K099, K100, S055, and S101). The experiment was set up in three replications in a randomized complete block design at two locations (Smolice and Kobierzyce) on plots of 10 m^2^. On average, there were 75 to 80 plants per plot. Morphological features were observed during the growing season and immediately after harvest. After harvest, the following yield structure traits were determined: ear length (cm), ear diameter (cm), kernel row length (cm), core length (cm), core diameter (cm), the number of rows, the number of kernels per row, and TSW (g). The grain yield (kg) from each plot was also analyzed. 

#### 2.2.2. Weather Conditions

**Figure 1:**
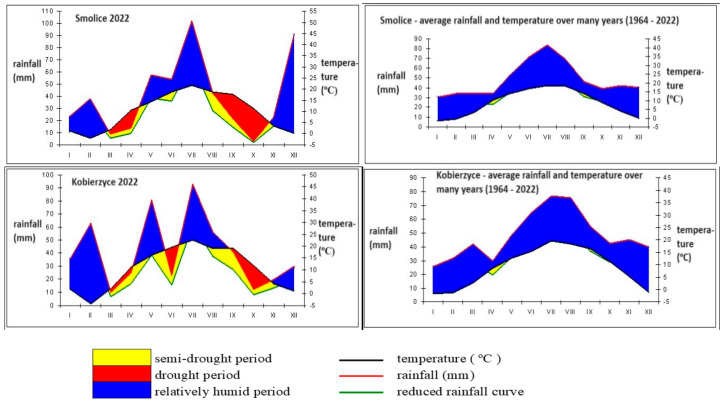
Temperature and rainfall in Smolice and Kobierzyce in 2022.

The data used came from the weather station belonging to the Poznań University of Life Sciences. In 2022, the average rainfall in Smolice was 39.94 mm, lower than the multi-year average rainfall, which amounted to 48.27 mm. The wettest month in this year was July (102 mm of rainfall), and the greatest drought was recorded in October (3 mm of rainfall). The temperature in this year ranged from −2.1 °C in February to 21.6 °C in July. The average air temperature in this year was higher than the long-term average air temperature by 1.05 °C and amounted to 9.79 °C. In the second decade of April, no rainfall was recorded, which had an adverse effect on corn emergence, while in May, rainfall totals were higher, which resulted in soil sealing and uneven corn emergence. In 2022, the average rainfall in Kobierzyce was 40.93 mm, and similarly to Smolice, it was lower than the multi-year average rainfall by 7.37 mm. The highest rainfall was recorded in May (81 mm), and the lowest was recorded in October (12.4 mm). The average air temperature in 2022 was 10.3 °C in Kobierzyce, higher than the long-term average temperature by 1.42 °C (Figure 1). The warmest month was July (22.5 °C), and the coldest was February (−4.3 °C). In 2022, no weather anomalies were observed in Smolice and Kobierzyce. Despite the periodic drought, the weather was typical for these areas of Poland. 

#### 2.2.3. DNA Isolation

The isolation of DNA from 66 inbred lines and 122 F_1_ hybrids was conducted using a commercial reagent kit purchased from Promega. The samples of isolated DNA were subjected to next-generation sequencing. DNA isolation from 20 reference genotypes was carried out using a commercial reagent kit purchased from A&A Biotechnology. The concentration and purity of the isolated DNA samples were determined using a DS-11 spectrophotometer from DeNovix. The isolated DNA template was adjusted to an equal concentration of 100 ng μL^−1^ by diluting it with double-distilled water (ddH_2_O).

#### 2.2.4. Genotyping

The methodology was taken from the work of Sobiech et al. [29]. DArTseq technology, which is based on next-generation sequencing, was applied for genotyping. The isolated DNA of the 188 maize plants tested (100 ng in 25 µL from each genotype) was sent in two 96-well Eppendorf plates for analysis to identify silicoDArT and SNPs. The analyses were performed at Diversity Arrays Technology, University of Canberra, Australia. Using methods proposed by Baird et al. [30], in the first step, the DNA template was digested with *Ape* KI, *Pst* I, and *Msp* I restriction enzymes to reduce genome complexity. The original GBS method used a single *Ape* KI enzyme (Elshire et al. [31]), and later, the method was expanded to include two additional enzymes: one infrequent cutter, *Pst* I, in combination with a frequent genomic DNA cutter, i.e., *Msp* I (Poland & Rife, [32]). Such an approach enabled the creation of a homogeneous library and the detection of most fragments associated with the infrequent cutting enzyme. A characteristic feature of the applied enzymes is their sensitivity to methylation, allowing for the filtering of non-coding regions and methylated repetitive sequences such as mobile elements. In the following step, genomic DNA fragments cleaved by restriction enzymes were ligated with adapters. Since the latter contains identifiers (so-called barcodes), the origin of each sample was strictly defined, and the identifiers met the appropriate criteria (Poland et al. [33]). The resulting PCR products were analyzed for size and constituted a genomic library, which was subsequently sequenced using a leading NGS platform (Kilian et al. [34]), Illumina, following the methodology detailed on the Diversity Arrays Technology’s website (https://www.diversityarrays.com/technology-and-resources/dartseq/) (URL accessed on 20 October 2023). 

#### 2.2.5. Association Mapping by Carrying Out a GWAS

Association mapping of 188 maize genotypes (66 lines and 122 hybrids) was performed for yield and yield structure traits by carrying out a GWAS. This mapping was conducted based on the results obtained from the genotyping and phenotyping analyses. The genotypic data were obtained from the DArTseq analysis, while the phenotypic data comprise results from field experiments concerning yield size and ear structure traits. The following yield structure traits were analyzed: ear length, ear diameter, core length, core diameter, the number of rows, the number of kernels per row, TSW, and yield per plot. Based on the GWAS, silicoDArT and SNP markers showing the highest significance level, i.e., those that were most strongly associated with yield structure traits and yield, were selected for further study.

#### 2.2.6. Physical Mapping 

Sequences of the silicoDArT and SNP markers, selected based on the GWAS, were subjected to BLAST (Basic Local Alignment Search Tool) analysis, which involved searching databases for sequences highly homologous to the selected silicoDArT and SNP markers. The following publicly available web browsers were used for this: CEPH Genotype database http://www.cephb.fr/en/cephdb/ (URL accessed on 20 October 2023), NCBI Map Viewer http://www.ncbi.nlm.nih.gov/projects/mapview/ (URL accessed on 20 October 2023), UCSC Genome Browser http://genome.ucsc.edu/ (URL accessed on 20 October 2023), Ensembl Map View http://ensembl.fugu-sg.org/common/helpview?kw=mapview;ref (URL accessed on 20 October 2023). The programs used helped identify the chromosomal locations of the retrieved sequences, similar to the analyzed sequences, and determine their physical location. The sequences of all genes located within the designated chromosomal region were further analyzed.

#### 2.2.7. Functional Analysis of Gene Sequences

Our functional analysis was carried out using the Blast2GO program https://www.blast2go.com/ (URL accessed on 20 October 2023). The sequences of all genes located in the chromosomal regions identified from the BLAST analysis were subjected to analysis. The aim was to obtain information about the biological function of the gene sequences located in a designated chromosomal region.

#### 2.2.8. Designing Primers for Identified SilicoDArT and SNPs Associated with Yield and Traits

The Primer 3 Plus program was used to design primers. The program can be accessed online and does not need to be downloaded or installed. Primer 3 Plus offers various options, ranging from various ways of specifying the sequence for which the primers are to be designed and general expectations regarding primer characteristics (size, the melting temperatures of both the primers and products, %GC, complementarity, etc.) to very detailed settings for primer parameters. 

#### 2.2.9. Polymerase Chain Reaction (PCR)

The identification of new markers linked to genes associated with the analyzed ear structure traits was carried out using a PCR. PCRs were conducted in a C1000 thermal cycler (Biorad, Hercules, CA, USA). The reaction mixture included the following: a 1 µ DNA template (50 ng µL^−1^), 4 µL polymerase buffer (5×), 1.6 µL dNTP (10 mM), 1.6 µL MgCl_2_ (25 mM), a 0.5 µL forward primer (10 µM), a 0.5 µL reverse primer (10 µM), 0.2 µL GoTaq polymerase (5 U µL^−1^), and 10.6 µL H_2_O. This composition was modified depending on the identified marker.

The PCR conditions were individually determined for each of the identified markers and differed in terms of primer annealing temperature, determined according to their respective melting temperatures. The following amplification temperature profile was used: initial denaturation for 5 min at 95 °C, followed by 35 cycles (denaturation for 45 s at 95 °C), primer annealing for 1 min (a different temperature was used for each pair of primers, consistent with their melting temperature), extension for 1 min at 72 °C, and final extension for 5 min at 72 °C before cooling to 4 °C. 

#### 2.2.10. Electrophoresis

The electrophoresis of the PCR products was conducted on a 2.5% agarose gel, with the addition of 1 µL of Midori Green solution, for 2 h at 100 V. The O’RangeRuler 50 bp (Fermentas, Waltham, MA, USA) was used as a reference to identify the sizes of the amplified products. The visualization of the separated DNA fragments was carried out under UV light and captured on digital images using the BIORAD gel visualization and documentation system.

## 3. Results

### 3.1. Field Experiment

**Figure 2:**
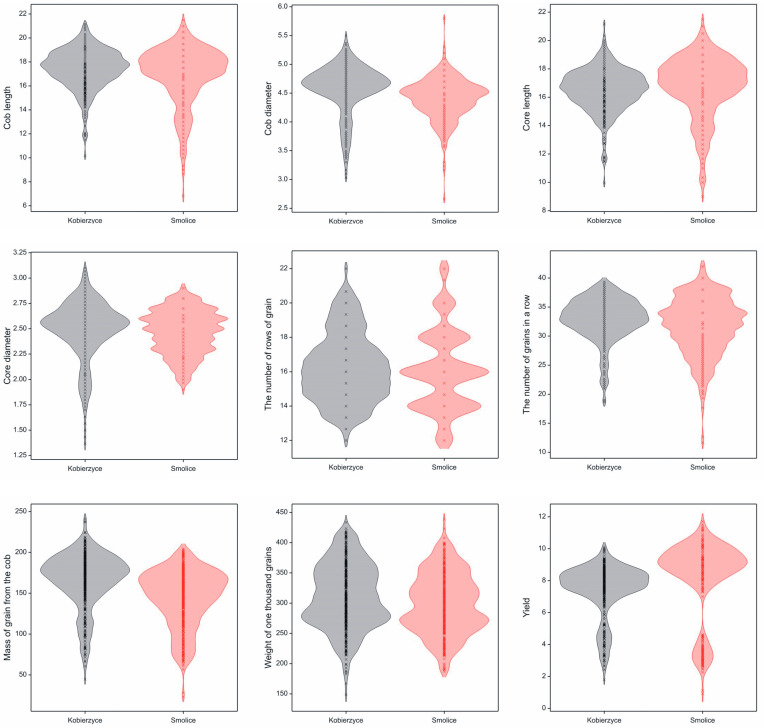
Density charts showing the distribution of the analyzed traits: cob length (cm), ear diameter (cm), core length (cm), core diameter (cm), the number of rows, the number of kernels per row, mass of grain per cob (g), TSW (g), and grain yield (kg).

The field experiment was established in two locations: Smolice (51°42′58.904″ N, 17°13′29.13″ E) and Kobierzyce (50°58′19.411″ N, 16°55′47.323″ E). This allowed us to perform and analyze biometric measurements of 188 maize genotypes. The measurement results were used for association mapping. After harvest, observations of the following yield structure traits were conducted: ear length, ear diameter, core length, core diameter, the number of rows, the number of grains per row, grain weight per ear, TSW, and yield per plot (Figure 2). Density plots were constructed to examine the distribution of all analyzed variables in both locations. The peaks in the density plots illustrate the ranges where the values of the analyzed traits are concentrated; e.g., for the majority of the analyzed genotypes in both locations (Smolice, Kobierzyce, Poland) ear length falls within the range of 17–19 cm. As demonstrated in the accompanying graphs, the distribution of the analyzed variables differed between the locations for core diameter, the number of rows, the number of grains per row, grain weight per ear, and yield per plot (Figure 2).

### 3.2. Phenotyping

An analysis of variance between the genotypes was performed for the recorded traits, and significant variation was observed for all traits. Our analysis of variance also showed statistically significant variation for all the studied traits between the locations where the field experiment was conducted. The line–location interaction was not significant, only being so for the number of rows (Table 1). 

**Figure 3:**
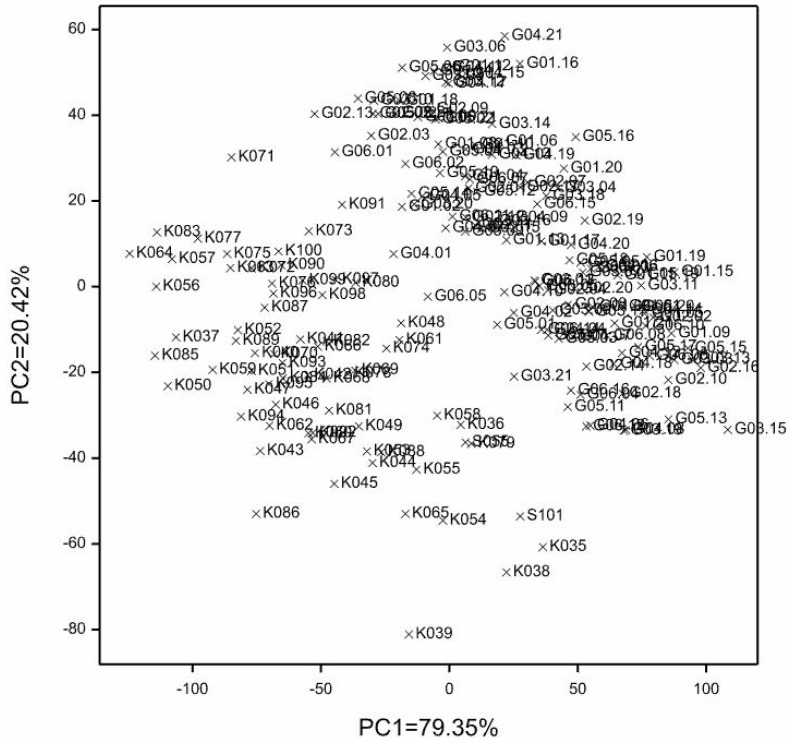
Canonical variate analysis for the studied traits.

To determine the relationships between groups of variables in the dataset, i.e., observations of the yield structure traits and yield per plot in both locations, a multivariate technique was applied, namely canonical variate analysis. All traits were characterized by a normal distribution. The grouping of genotypes into lines and hybrids could be observed (Figure 3).

**Figure 4:**
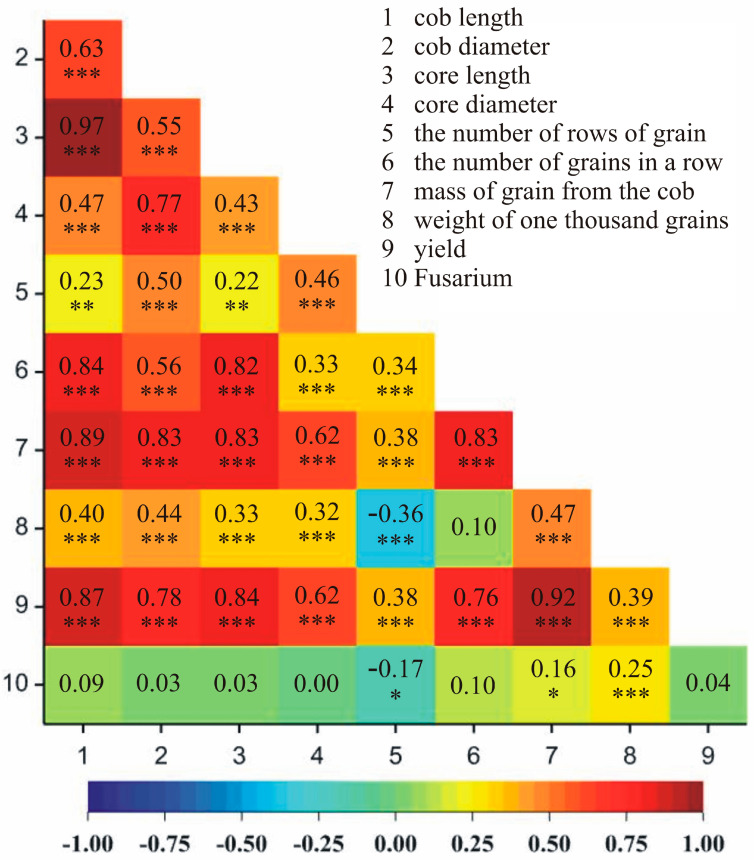
Heat map showing correlations between analyzed yield structure traits in Smolice. * *p* < 0.05, ** *p* < 0.01, *** *p* < 0.001.

**Figure 5:**
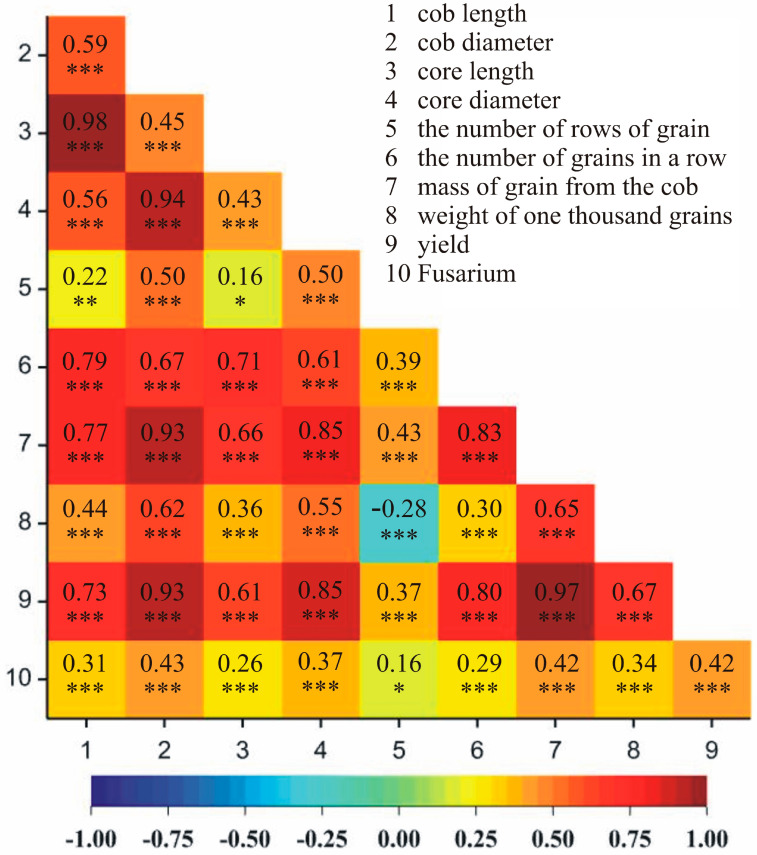
Heat map showing correlations between analyzed yield structure traits in Kobierzyce. * *p* < 0.05, ** *p* < 0.01, *** *p* < 0.001.

Correlations between the observed traits were analyzed in both locations, i.e., Smolice and Kobierzyce. It was demonstrated that in Smolice, the most strongly positively correlated traits were cob length and core length (97%), mass of grain from the cob and yield (92%), cob length and mass of grain from the cob (89%), and cob length and yield (87%) (Figure 4). In the case of Kobierzyce, the following traits were strongly positively correlated: cob and core length (98%), mass of grain from the cob and yield (97%), cob and core diameter (94%), cob diameter and mass of grain from the cob (93%), and cob diameter and yield (93%) (Figure 5). 

### 3.3. DNA Isolation 

DNA isolation from the 188 genotypes which were sent for next-generation sequencing was performed using a kit from Promega. The yield from individual isolations was high, ranging from 106 ng/μL for line 15 to 935.24 ng/μL for line 34. The purity of the isolated DNA was very good and averaged 1.8 for absorbance A260/A280. The exception were two samples: line 17, which had a purity of 1.54, and line 64, which had a purity of 2.39. Given the relatively high concentration of DNA obtained, the samples were adjusted to a uniform concentration of 100 ng μL^−1^, required for next-generation sequencing analyses.

### 3.4. Genotyping

A total of 92,614 molecular markers were obtained as a result of next-generation sequencing, including 60,436 SilicoDArT markers and 32,178 SNPs. MAF > 0.25 and a number of missing observations <10% were applied as criteria to determine the usefulness of the identified markers. This operation reduced the number of markers to 32,900 (26,234 DArTs and 6666 SNPs), which were subsequently used for association mapping (Table 2). The majority of SNP and Silico DArT markers were associated with yield (18,352—Kobierzyce and 18,751—Smolice), mass of grain from the cob (17,685—Kobierzyce and 18,314—Smolice), and core diameter (17,787—Kobierzyce and 16,018—Smolice). Few markers were associated with the number of rows of grain (12,757—Kobierzyce and 11,714—Smolice) and the number of grains in row (13,265—Kobierzyce and 13,981—Smolice) (Table 2). In order to narrow down the number of markers for physical mapping, 20 markers were selected from among all the significant ones that were associated with the same traits in both locations (Kobierzyce and Smolice). 

**Figure 6:**
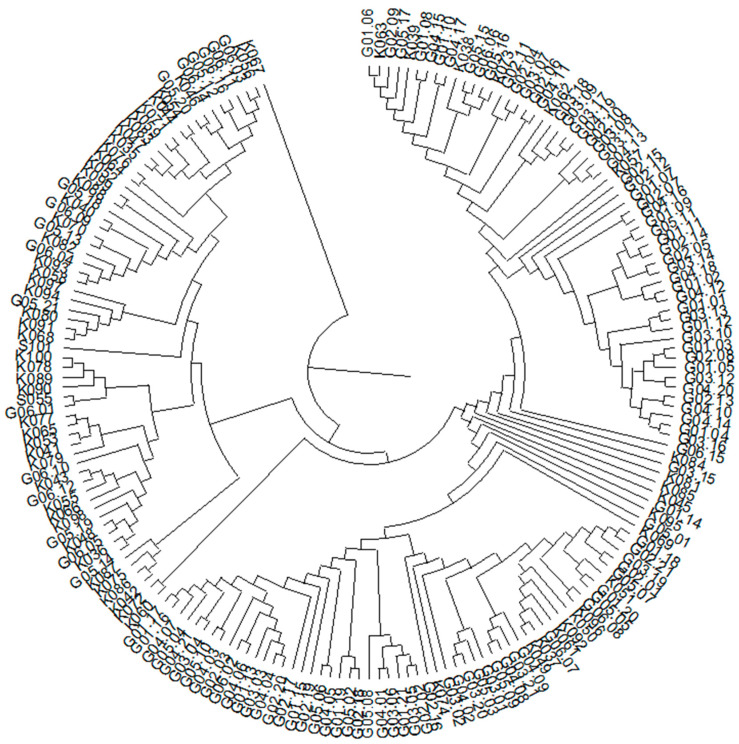
Dendrogram showing the genetic similarity between the analyzed genotypes.

Based on the identified SNP and SilicoDArT molecular markers, a dendrogram of genetic similarity was constructed for the 188 analyzed genotypes (Figure 6). The dendrogram very clearly shows two distinct similarity groups. The first group consisted of 65 inbred lines from HR in Kobierzyce, while the second group included 122 analyzed hybrids and 1 inbred line. Such an ideal clustering demonstrates the usefulness of SNP and silico DArT markers for grouping genotypes by genetic similarity. 

A total of 20 of the 32,900 markers (26,234 DArTs and 6666 SNPs) significantly associated with the analyzed yield structure traits and yield were selected. These markers were significant for the same traits in both locations (Kobierzyce and Smolice) (Table 3). An attempt was also made to determine the location of selected SNP markers. Unfortunately, it was not possible to determine the position of one marker. The next step was to design primers for the identification of the 19 selected and localized markers. After determining the location of the 19 selected SNPs, an attempt was made to design primers for their identification. Primer sequences are shown in Table 4. 

### 3.5. The Identification of New Molecular Markers Associated with Yield and Yield Structure Traits Using Polymerase Chain Reaction (PCR)

**Figure 7:**
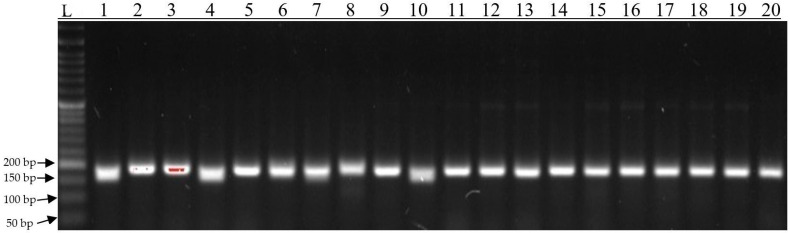
Electropherogram showing the amplification products of 189 bp, characteristic of the SNP28629 marker. L-ladder, 1–20 genotypes.

**Figure 8:**
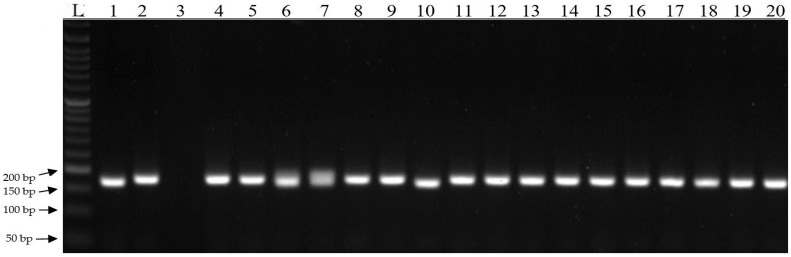
Electropherogram showing the amplification products of 189 bp, characteristic of the SNP 29294 marker. L-ladder, 1–20 genotypes.

Of the 19 markers selected, 2 (28629 and 29294) produced different amplification products on the electropherograms. The first 10 genotypes based on field observations are classified as the highest yielding while genotypes numbered 11–20 are classified as the lowest yielding. For marker 28629, a specific product of 189 bp was observed for genotypes 1, 4, and 10. Non-specific products of 200 bp were obtained for the remaining genotypes (Figure 7). This marker is located on chromosome 8, 3130 bp upstream of “protein senescence-associated gene 21, mitochondrial” and 91 bp downstream of “uncharacterized protein loc100382335”. For marker 29294, a specific product of 189 bp was observed for genotypes 1 and 10. Non-specific products of 200 bp were obtained for the remaining genotypes (Figure 8). This marker is located on chromosome 5 within the hydroxyproline o-galactosyltransferase galt6 gene. Both markers will undergo further testing on a larger number of extreme genotypes to be used for the initial selection of high-yielding genotypes.

## 4. Discussion

The breeding of heterozygous maize varieties consistently aims to harness the potential of hybrid vigor, shorten the breeding process (e.g., by utilizing doubled haploid lines), and improve and reduce the costliness of seed production. The priority for all breeders is to obtain high-yielding and disease-resistant maize varieties [35].

In the present study, an analysis of variance was performed based on phenotypic observations (related to yield and yield structure traits). For all traits, significant variation was observed between the genotypes. Our analysis of variance also showed statistically significant variation for all studied traits between locations where the field experiment was established. The interaction of line and trial location was not significant only for the number of rows. To determine the relationships between the groups of variables in the dataset, i.e., observations of the yield structure traits and yield per plot in both locations, a multivariate technique was applied, namely canonical variate analysis. All traits were characterized by a normal distribution. The grouping of genotypes into lines and hybrids could be observed. 

Phenotypic analysis, unfortunately, does not allow for the selection of parental components for heterosis crosses because traditional methods used in heterosis breeding are insufficient in the era of technological progress. In light of this challenge, modern agriculture has led to the harnessing of high-throughput techniques for analyzing the genomes of crop plants for their subsequent use in improving existing varieties, including maize [36]. Such a genomics-focused approach allows one to obtain information about coding regions that provide information on protein structure (genomic), as well as intergenic regions; both types can be successfully applied to improve crop plant varieties [37].

The introduction of next-generation sequencing (NGS) methods has enabled the elucidation of nucleotide sequences in plants other than model organisms such as *Arabidopsis thaliana*, which are characterized by a small genome. Crop species of interest mainly include maize, coffee, or sugarcane [38]. 

In recent years, many authors have attempted to identify molecular markers linked to functionally important traits in maize. Bocianowski et al. [39] used NGS technology and associative mapping to identify markers related to the heterosis effect in maize. Using the same methods, Sobiech et al. [40] identified markers linked to the resistance of maize plants to fusarium. In turn, Tomkowiak et al. [41] identified six SNP markers (1818; 14506; 2317; 3233; 11657; 12812) located inside genes, on chromosomes 8, 9, 7, 3, 5 and 1, related to the amount of yield in corn. The authors of [42] identified four genes—sucrose synthase 4 isoform ×2 gene, phosphoinositide phosphatase sac7 isoform ×1 gene, putative SET domain containing protein family isoform ×1 gene, and grx_c8–glutaredoxin subgroup iii—which can significantly regulate the level of seed vigor and germination of maize seeds.

In the present study, a total of 92,614 molecular markers were obtained utilizing next-generation sequencing, including 60,436 SilicoDArT markers and 32,178 SNPs. MAF > 0.25 and a number of missing observations <10% were applied as criteria to determine the usefulness of the identified markers. In this way, 32,900 markers (26,234 DArTs and 6666 SNPs) were obtained and applied for association mapping. 

NGS technology is utilized for sequencing genomes and transcriptomes, studying protein–DNA/RNA interactions, assessing methylation levels, discovering new DNA polymorphisms, and conducting meta-genomic studies [43]. This technology allows for the analysis of various DNA fragments represented by multiple copies during a single reaction, library preparation, and the subsequent collection of gigabases of genomic data from a single sequencing run [44,45]. This not only increases the number of samples examined but also enhances the reliability of the obtained sequencing results. This is particularly valuable when the variation between specific genotypes is small [36]. The costs and time required for sequencing reactions, when calculated per unit of obtained information, are significantly lower compared to the costs of analyses conducted using traditional capillary sequencers [46]. 

Another sequencing strategy, primarily used to study the interactions between plants and their environment, is the use of NGS methods to characterize the plant transcriptome in different physiological states. The analysis of cDNA sequences provides information about expressed sequence tags (ESTs), which are transcribed in specific tissues and organs, and despite some limitations, these data are very useful for breeders [36,47,48]. Next-generation sequencing techniques also enable qualitative and quantitative analyses of genes expressed under different conditions, and the results of these analyses are used for association mapping [49,50,51,52].

In the present study, association mapping was carried out, and it was found that the highest number of SNPs and silico DArT markers were associated with yield per plot (18,352—Kobierzyce and 18,751—Smolice), grain weight per ear (17,685—Kobierzyce and 18,314—Smolice), and core diameter (17,787—Kobierzyce and 16,018—Smolice). The fewest markers were associated with the number of rows (12,757—Kobierzyce and 11,714—Smolice) and the number of grains per row (13,265—Kobierzyce and 13,981—Smolice). To narrow down the number of markers for physical mapping, 19 markers were selected from among all significant ones that were associated with the same traits in both locations (Kobierzyce and Smolice). These markers were tested on high- and low-yielding reference genotypes. As a result of testing, two markers (28629 and 29294) that differentiated the tested genotypes were selected. For marker 28629, a specific product of 189 bp was observed for genotypes 1, 4, and 10. For marker 29294, a specific product of 189 bp was observed for genotypes 1 and 10. 

Thanks to their specific features, SNP and SilicoDArT markers find many applications, including in the creation of molecular linkage maps and the identification of quantitative trait loci (QTLs) responsible for the inheritance of quantitative traits. Additionally, they are used for origin analysis, fingerprinting of cultivated varieties, in studies on population genetic diversity and gene flow, and plant evolutionary genetics [52].

In the present study, we identified 19 SNP markers that are significantly associated with yield structure traits in maize. Five of these markers (28629, 28625, 28640, 28649, and 29294) are located within genes that can be considered candidate genes related to yield traits. Marker 28629 is located on chromosome 8 within the leucine-rich repeat receptor-like protein kinase gene. Receptor-like kinases (RLKs) are a diverse group of transmembrane proteins characterized with a ligand-binding domain to receive signal molecules, a membrane-spanning domain to anchor the protein, and a cytoplasmic protein kinase domain to transduce signals downstream [53]. According to reports in the literature, the first RLK was isolated from maize, and then numerous RLKs were identified in over 20 plant species [54]. RLKs can indirectly influence the yield of maize because they mediate many signaling messages on the cell surface and act as key regulators during developmental processes [55,56,57]. Genetic and biochemical studies conducted by other scientists have also shown that plant LRR-RLKs play an important role in various processes during growth and development [58,59]. CLV and RPK2 have also been found to be essential receptor-like kinases in the formation and maintenance of the shoot apical meristem [60,61]. Another significant marker was SNP 28625, located on chromosome 1 within the arabinosyltransferase (arad1) gene. As reported in [62], gene-encoding arabinosyltransferase ARAD1 catalyzes the polymerization of arabinose into the arabinan of arabinogalactan during secondary wall formation in loblolly pine. Research indicates a connection between arabinogalactan proteins and lignin biosynthesis for cell wall formation. It is well known that lignin occurs in the cell wall and is necessary for the transport of water and aqueous nutrients in plant stems. The polysaccharide components of plant cell walls are hydrophilic and therefore permeable to water, while lignin is hydrophobic. The cross-linking of polysaccharides by lignin prevents the absorption of water by the cell wall. Consequently, lignin enables the plant’s vascular tissue to conduct water efficiently, which is very important for the proper growth and development of the plant and its yield [63]. Therefore, there is a high probability that the ARAD1 arabinosyltransferase gene may influence the yield of maize. Research conducted in recent years has shown that cell walls can play an important role in intercellular communication, not only as a pathway for the transport of signaling molecules, or as an area of the cell in which the receptor domains of membrane proteins operate, but also as a source of signals that influence the functioning of cells [64,65,66]. The third significant SNP (28640) was located on chromosome 9 inside the sugar phosphate gene. Sucrose phosphate synthase (SPS) is a key enzyme in the sugar metabolic pathways in plants. SPS catalyzes the conversion of fructose-6-phosphate and uridine diphosphate-glucose (UDP-glucose) into sucrose-6-phosphate which is a substrate in the synthesis of sucrose [67,68]. SPS exists in many isoforms which may play various functional roles and are specific to different tissues and stages of development. Particularly, knowledge about the relationship of the localization of the individual forms of these enzymes and their role in plant responses to various stresses is highly desirable. It has been demonstrated, for instance, that several maize SPS sequences are most strongly expressed in the leaves and less intensively in pollen and kernels, and this is related to reactions to different abiotic factors [69]. SUS isoforms have been found in different areas of cell walls [70,71]). In maize, the specificity of the function of individual SUS isoforms in both cytoplasmic and membrane-associated sucrose degradation was emphasized in [72]. The fourth significant SNP (28649) was located on chromosome 4 within the gene annotated as ubiquitin carboxyl-terminal hydrolase 15 isoform x2. Ubiquitin carboxyl-terminal hydrolases (UCHs) belong to an enzymatic subclass of deubiquitinating enzymes (DUBs). The function of this gene in maize has not been described in the literature. The fifth significant SNP (29294) was located on chromosome 5 within the hydroxyproline o-galactosyltransferase galt6 gene. According to our analyses and data in the literature, this gene may be related to the yield of maize because, according to Kaur et al. (2023), hydroxyproline o-galactosyltransferase galt6 plays an important role in various stages of plant growth and development [73]. Moreira et al. (2023) have reported that arabinogalactan proteins (AGPs) are hydroxyproline-rich, sugar-rich glycoproteins widely distributed in the plant kingdom [74]. The synthesis of their complex carbohydrates is initiated by a family of hydroxyproline galactosyltransferase (Hyp-GALT) enzymes, which add the first galactose to Hyp residues in the protein backbone.

## 5. Conclusions

In the present study, we identified 19 SNP markers that are significantly associated with yield structure traits in maize. Five of these markers are located within genes that can be considered candidate genes related to yield traits: marker 28629, located on chromosome 8 within the leucine rich repeat receptor like protein kinase gene; marker 28625, located on chromosome 1 within the arabinosyltransferase (arad1) gene; marker 28640, located on chromosome 9 inside the sugar phosphate gene; marker 28649, located in ubiquitin carboxyl-terminal hydrolase 15 isoform x2ubiquitin carboxyl-terminal hydrolase 15 isoform x3; marker 29294, located in hydroxyproline o-galactosyltransferase galt6. These genes will be subjected to expression analysis in the next stage of our research. The identified markers were tested on high- and low-yielding reference genotypes. The testing was aimed at selecting markers that could be used to select high-yielding genotypes. In the case of two markers (28629 and 29294), different amplification products were obtained on the electrophorograms. For marker 28629, a specific product of 189 bp was observed in genotypes 1, 4, and 10. For marker 29294, a specific product of 189 bp was observed in genotypes 1 and 10. Both markers will be further tested on more extreme genotypes so that they can be applied in the preliminary selection of high-yielding genotypes.

## Figures and Tables

**Table 1:** *F*-statistics from our analysis of variance for the analyzed yield structure traits.

Trait	Analysis of Variance	
	*F*-Statistics	*F* pr.
Cob length	20.84	<0.001
Cob diameter	24.10	<0.001
Core length	21.02	<0.001
Core diameter	22.65	<0.001
The number of rows of grain	11.17	0.569
The number of grains in row	16.07	<0.001
Mass of grain from the cob	15.78	<0.001
Weight of one thousand grains	14.31	<0.001
Yield per plot	50.16	<0.001

**Table 2:** SilicoDArT and SNP molecular markers significantly associated with analyzed yield structure traits in Kobierzyce (K) and Smolice (S) (significant associations selected at *p* < 0.001 with Benjamini–Hochberg correction for multiple testing).

Trait		Cob Length		Cob Diameter		Core Length		Core Diameter		The Number of Rows of Grain		The Number of Grains in a Row		Mass of Grain from the Cob		Weight of One Thousand Grains		Yield	
Location		K	S	K	S	K	S	K	S	K	S	K	S	K	S	K	S	K	S
Number of significant markers	DArT	10,209	14,172	14,404	12,934	9189	13,542	14,254	10,584	10,054	9286	10,768	11,342	14,315	14,816	11,205	11,688	14865	15,173
	SNP	2356	3343	3383	3084	2156	3139	3330	2626	2703	2428	2497	2639	3370	3498	2631	2857	3487	3578
	Total	12,565	17,515	17,787	16,018	11,345	16,681	17,584	13,210	12,757	11,714	13,265	13,981	17,685	18,314	13,836	14,545	18,352	18,751
Minimal effect	DArT	−1.913	−3.579	−0.693	−0.482	−1.49	−3.00	−0.447	−0.237	−1.82	−1.991	−4.378	−5.718	−49.63	−53.66	−60.49	−48.04	−2.67	−4.23
	SNP	−1.885	−3.148	−0.5575	−0.398	−1.432	−2.94	−0.378	−0.212	−2.01	−1.965	−4.228	−5.602	−43.60	−45.43	−60.26	−47.82	−2.31	−3.59
	Total	−1.913	−3.579	−0.693	−0.482	−1.49	−3.00	−0.447	−0.237	−2.01	−1.991	−4.378	−5.718	−49.63	−53.66	−60.49	−48.04	−2.67	−4.23
Maximum effect	DArT	2.815	5.174	0.9446	0.5911	2.032	4.406	0.613	0.286	2.665	2.892	6.926	8.191	74.59	72.63	90.47	66.54	3.912	5.911
	SNP	2.788	5.183	0.9493	0.6064	2.061	4.435	0.620	0.295	2.679	2.648	6.909	8.217	74.92	72.63	89.84	67.12	3.918	5.911
	Total	2.815	5.183	0.9493	0.6064	2.061	4.435	0.620	0.295	2.679	2.892	6.926	8.217	74.92	72.63	90.47	67.12	3.918	5.911
Medium effect	DArT	1.702	2.576	0.466	0.316	1.305	2.269	0.304	0.159	0.691	1.344	4.180	4.820	36.808	35.875	52.981	24.559	1.898	2.822
	SNP	1.867	2.739	0.493	0.335	1.423	2.430	0.326	0.169	0.708	1.303	4.452	5.013	39.060	37.700	57.152	27.142	2.012	2.993
	Total	1.733	2.607	0.471	0.320	1.328	2.299	0.308	0.161	0.695	1.335	4.232	4.857	37.237	36.223	53.774	25.067	1.920	2.855
Total effect	DArT	17,375	36,504	6706	4089	11,994	30,729	4328	1685	6952	12,476	45,014	54,673	526,910	531,519	593,654	287,051	28,211	42,819
	SNP	4399	9158	1669	1035	3068	7627	1086	443	1915	3164	11,118	13,228	131,632	131,874	150,368	77,546	7017	10,709
	Total	21,774	45,662	8375	5124	15,062	38,357	5413	2128	8867	15,640	56,131	67,901	658,542	663,394	744,022	364,597	35,227	53,528

**Table 3:** Characteristics and locations of markers significantly associated with the analyzed traits.

Marker	Type	Chromosome	Candidate Genes
28629	SNP	chr. 8	probable leucine-rich repeat receptor-like protein kinase
28630	SNP	chr. 8	637 bp at 5′ side: putative protein phosphatase 2c 4658414 bp at 3′ side: metallothionein-like protein type 2
28631	SNP	chr. 4	99861 bp at 5′ side: uncharacterized protein loc1002769903113 bp at 3′ side: disease resistance protein rpm1 isoform x1
28632	SNP	chr. 7	uncharacterized atp-dependent helicase ypra
28633	SNP	chr. 7	4486 bp at 5′ side: lon protease homolog 2, peroxisomal54848 bp at 3′ side: uncharacterized protein loc100280671
28634	SNP	chr. 7	132577 bp at 5′ side: isoamylase-type starch debranching enzyme iso3 isoform x135136 bp at 3′ side: uncharacterized protein loc100272620
31977	SNP	chr. 1	1394 bp at 5′ side: 60 s ribosomal protein l32-like68287 bp at 3′ side: uncharacterized protein loc100193765 isoform x1
29503	SNP	chr.4	uncharacterized protein loc103655564
28625	SNP	chr. 1	probable arabinosyltransferase arad1
28640	SNP	chr. 9	probable sugar phosphate/phosphate translocator
28648	SNP	chr. 1	uncharacterized protein loc100502264
28639	SNP	chr. 8	3130 bp at 5′ side: protein senescence-associated gene 21, mitochondrial91 bp at 3′ side: uncharacterized protein loc100382335
28649	SNP	chr. 4	ubiquitin carboxyl-terminal hydrolase 15 isoform x2ubiquitin carboxyl-terminal hydrolase 15 isoform x3
28654	SNP	chr. 5	318 bp at 5′ side: uncharacterized protein loc100383290 isoform x12155 bp at 3′ side: uncharacterized protein loc111589274
30773	SNP	chr.4	30080 bp at 5′ side: uncharacterized protein loc103653173940 bp at 3′ side: uncharacterized protein loc100193686 isoform x1
30772	SNP	chr. 3	1814 bp at 5′ side: uncharacterized protein loc100192805 isoform x240087 bp at 3′ side: uncharacterized protein loc100278501
29294	SNP	chr. 5	hydroxyproline o-galactosyltransferase galt6
28262	SNP	chr. 9	uncharacterized protein loc100383550uncharacterized protein loc100383550 isoform x1
28263	SNP	chr. 3	uncharacterized protein loc103650272

**Table 4:** Sequences of the designed primers used to identify the newly selected markers significantly associated with the analyzed traits.

Marker	Primer Sequences		Size of the Product	Tm [°C]
	Forward	Reverse		
28629	CACCTGGAGAGGCCCTGCAG	TGAAGACTGGACTCGGTCGC	437	61
28630	AGGGACAACATACACTGCAG	ACGGTAAAGAGACACAATTCCCT	189	58
28631	AAATCTGCGATCAACTGCAG	GACTGGAGAGCCAGAACCTG	291	55
28632	AGAGAAGTATTGTCTTTCTGCAG	GCTGTCCTTGATGCCAAGTC	437	57
28633	GATTATCCTTCGAGCTGCAG	ACATGAATGTTGCAGGCAGG	423	61
28634	TTTATTCCTCACTGCTGCAG	AGAAAGAAGGAATGTAACAACACG	342	56
31977	CCACTTGCTCTCTGCTGCAG	CTCTCTATGGCTCGTCGCTG	207	59
29503	ACTCAGTAGCAGCACTGCAG	GTAGCTGCTGCCTCTTCCAA	455	59
28625	GCGCGCTTGTGAAGCTGCAG	TTTCAGGGCGGGAAGGTTCG	488	56
28640	TACTCTGTTAACACCTGCAG	ATGCGCAGTTGCCTACTTAT	158	62
28648	AATAAGAGCTTTTGCTGCAG	AGCGACGAGTAATCAATCCC	453	54
28639	GGCTTCCAGCTTGGCTGCAG	ATACGTGACGCACGAGACAAAC	189	58
28649	TGGAGCAAAAGTATCTGCAG	TTTTTCACCTCTTGACGGGC	471	61
28654	GGAATCCATCAGTTCTGCAG	CTTCCCCGAGTGCATATCCT	169	59
30773	AAACCAAACCGCCGCTGCAG	CAGGCAGAGCTCAGTCCGAA	411	61
30772	GCATTTTACGGGGTCTGCAG	AGAGCTTGCTCCCTTGAACG	500	57
29294	GTTTGTGGGACAAACTGCAG	AGGGCTTAATTTATTTCCAGCCA	184	57
28262	TAAGAATGAGAAGCCTGCAG	GAATGCACTGTTGTTCTGCC	454	59
28263	GCCGGGAATAACTTCTGCAG	GTTCTTACTTCCGCCAGGCT	358	59

## Data Availability

Data are contained within the article.
